# Effects of environmental enrichment on lymph node mitochondrial-redox status and immune response in adult male C57BL/6 mice

**DOI:** 10.1038/s41598-026-55690-8

**Published:** 2026-06-03

**Authors:** Matheus Santos de Sousa Fernandes, Alexandre Kanashiro, Débora Eduarda da Silva Fidélis, Tiago Lacerda Ramos, Burak Yagin, Fatma Hilal Yagin, Vanessa Lima de Souza, Claudia Jacques Lagranha, Monira I. Aldhahi, Fabrício Oliveira Souto

**Affiliations:** 1 Keizo Asami Institute, Federal University of Pernambuco, Recife, Pernambuco Brazil; 2 Postgraduate Program in Biology Applied to Health, Federal University of Pernambuco, Recife, Pernambuco Brazil; 3 Department of Pharmacology, Ribeirao Preto Medical School of the University of Sao Paulo, Ribeirao Preto, SP Brazil; 4 Center for Research in Inflammatory Diseases, Ribeirao Preto Medical School of the University of Sao Paulo, Ribeirao Preto, SP Brazil; 5 Department of Biostatistics and Medical Informatics, Faculty of Medicine, Inonu University, 44280 Malatya, Turkey; 6 Department of Biostatistics, Faculty of Medicine, Malatya Turgut University, 44210 Malatya, Turkey; 7 Laboratory of Biochemistry and Molecular Biology of Physical Exercise, Federal University of Pernambuco, Vitória de Santo Antão, Pernambuco Brazil; 8 Department of Rehabilitation Sciences, College of Health and Rehabilitation Sciences, Princess Nourah bint Abdulrahman University, P.O. Box 84428, 11671 Riyadh, Saudi Arabia

**Keywords:** Environmental enrichment, Lymph node, Redox status, Blood cytokines, Inflammation, Biochemistry, Immunology, Neuroscience, Physiology

## Abstract

Environmental enrichment (EE), an experimental paradigm promoting cognitive, sensory, social, and motor stimulation, has been associated with neuroprotective effects. Although EE modulates immune function, its influence on the basal redox and metabolic environment of lymph nodes remains unclear. This study investigated whether EE induces physiological immunometabolic and redox adaptations in lymph nodes under non-pathological conditions. Adult male C57BL/6 mice were housed for six weeks in either a standard environment (SE) or EE, followed by evaluation of mitochondrial markers, redox balance, antioxidant defenses in axillary lymph nodes, and systemic basal cytokine profiles. EE did not modify citrate synthase activity, indicating preserved mitochondrial content. Both NAD^+^ and NADH levels increased significantly, while the NAD^+^/NADH ratio remained unchanged. EE reduced lipid peroxidation without affecting protein oxidation and selectively increased catalase activity, whereas superoxide dismutase and glutathione S-transferase activities were unchanged. Non-enzymatic antioxidant defenses were enhanced, with increased reduced glutathione (GSH), and an elevated GSH/GSSG ratio, despite reduced total sulfhydryl levels. EE did not alter most circulating cytokines but selectively reduced interferon-γ (IFN-γ) levels. Overall, EE promoted a redox-optimized and metabolically resilient lymph node environment, characterized by enhanced antioxidant buffering capacity, without inducing broad immune activation or suppression.

**Supplementary Information:**

The online version contains supplementary material available at 10.1038/s41598-026-55690-8.

## Introduction

Environmental enrichment (EE) is a well-established experimental paradigm characterized by enhanced social interaction, cognitive stimulation, and voluntary physical activity, designed to model biologically relevant ecological complexity. In the field of neuroscience, EE has been demonstrated to enhance learning, memory, and attention, while also providing resilience against stress-related neuropsychiatric disorders and neurodegenerative diseases, such as Alzheimer’s and Parkinson’s disease^1–5^.

Beyond the central nervous system (CNS), EE has emerged as a systemic modulator of immune function. Experimental evidence indicates that EE influences cytokine production, immune cell activation, and stress-induced immune dysregulation, supporting its role in maintaining immune homeostasis^6–8^. However, despite these advances, the impact of EE on lymph nodes, the primary anatomical sites of adaptive immune activation, remains poorly characterized.

Lymph nodes provide the metabolic and redox framework required for antigen presentation, T- and B-cell activation, clonal expansion, and effector differentiation. These processes are energetically demanding and highly sensitive to redox balance, which regulates mitochondrial metabolism, intracellular signaling thresholds, and cytokine production. Disruption of redox homeostasis within lymphoid tissues promotes oxidative stress, exaggerated Th1 polarization, and excessive interferon-gamma (IFN-γ) production, features commonly associated with aging, autoimmune disease, and chronic inflammatory states^9^.

The redox balance in immune tissues is maintained through coordinated regulation of mitochondrial metabolism, enzymatic antioxidant systems, and the glutathione redox buffer. EE has been shown to enhance antioxidant defenses, reduce oxidative damage, and improve metabolic efficiency in peripheral organs such as skeletal muscle, liver, and heart^10–14^. Given the central role of oxidative stress in immunosenescence and inflammatory disease, EE represents a compelling non-pharmacological strategy capable of inducing adaptive, homeostatic redox responses^2,11,12,14^.

Despite this, whether EE alters the basal redox and metabolic environment of lymph nodes under non-pathological conditions remains unknown. Addressing this gap is essential for understanding how environmental factors shape immune function at the tissue level. Therefore, the present study investigated the effects of EE on mitochondrial redox indicators, antioxidant defense systems, and systemic cytokine profiles in the lymph nodes of adult male mice.

## Materials and methods

### Ethical procedures

This study was approved by the Ethics Committee on the Use of Animals of the Federal University of Pernambuco (CEUA-UFPE) under registration number 0051/2023.

All experimental procedures involving animals followed the ARRIVE guidelines and were conducted in compliance with the UK Animals (Scientific Procedures) Act 1986 and its related regulations, the EU Directive 2010/63/EU concerning the protection of animals used for scientific research, and the National Research Council’s Guide for the Care and Use of Laboratory Animals.

### Animals and experimental design

In this study, male C57BL/6 mice (n = 14) obtained from the breeding and experimental vivarium of the Keizo Asami Institute at the Federal University of Pernambuco (ILIKA/UFPE) were used. The animals were maintained under controlled conditions at 22 ± 2 °C, with a 12 h light/dark cycle, and had ad libitum access to water and standard commercial chow (Nuvilab CR-1, Quimtias, Brazil). When the animals reached 45 days old, they were randomly assigned based on their specific environmental conditions. Subsequently, they were split into two groups: one for the standard environment (SE) (n = 7; body weight: 25.50 g ± 1.71 g) and another for the EE (n = 7; body weight: 25.00 g ± 2.03 g).

### Environmental enrichment (EE) protocol

**Fig. 1:**
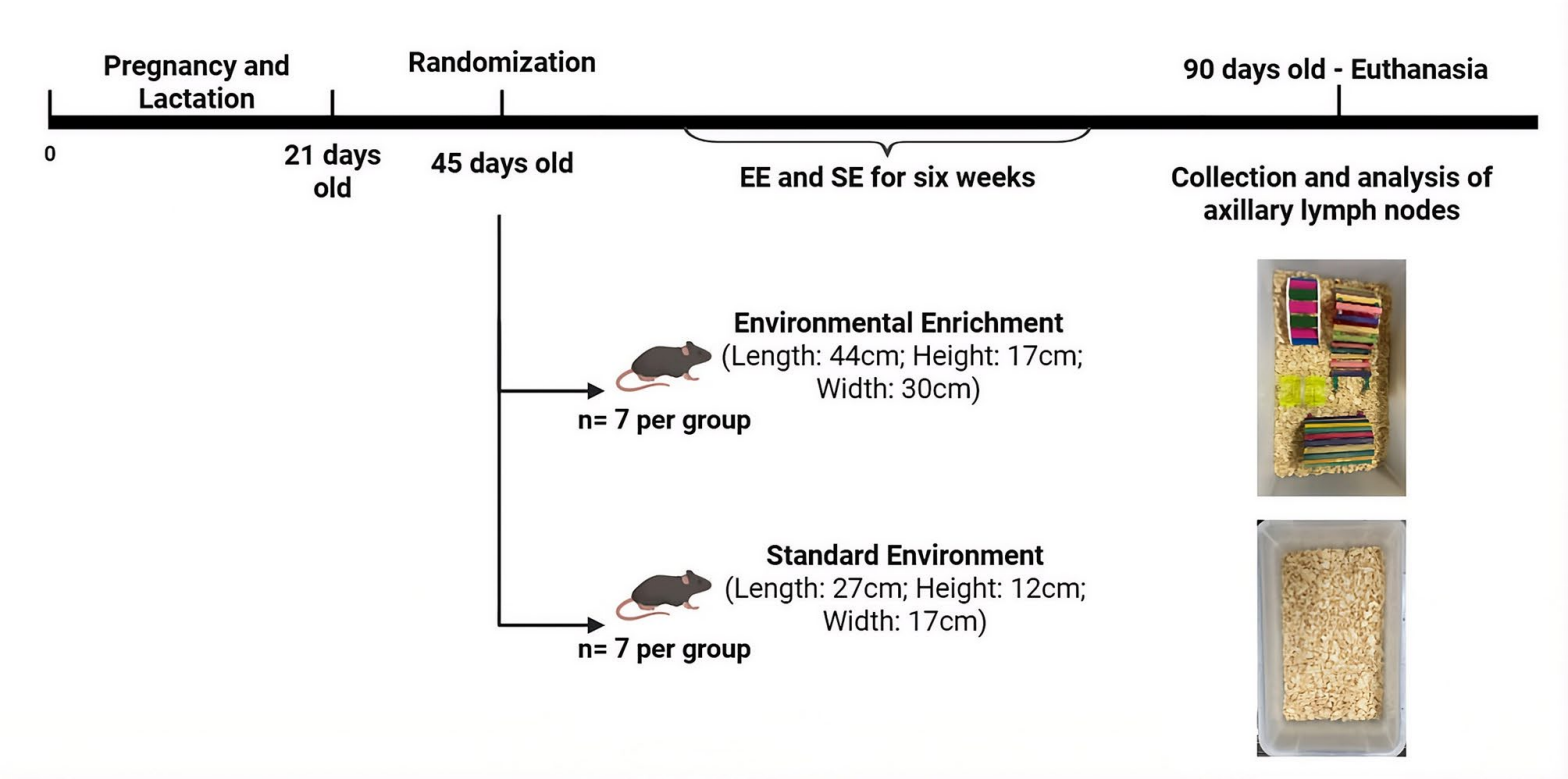
Experimental design of a 6-week Environmental Enrichment protocol in male C57BL/6 mice, with collection of axillary lymph nodes for analysis.

To establish EE, the model was first standardized for C57BL/6 mice. This strategy involved introducing inanimate objects to stimulate sensory, cognitive, and motor activities. Furthermore, in accordance with protocols widely reported in the literature, enriched housing conditions are recommended to accommodate a higher number of animals and to provide larger cage dimensions—length (L), width (W), and height (H)—than those used in standard housing, thereby promoting greater opportunities for social interaction. In this study, a range of enrichment materials was utilized, including plastic items (e.g., tunnels, ladders, and toys), wooden structures (e.g., shelters and toys), and running wheels. Cage dimensions were as follows: EE: 44 cm (L) × 30 cm (W) × 17 cm (H); SE: 27 cm (L) × 17 cm (W) × 12 cm (H). To enhance environmental complexity and variability, both the number and spatial arrangement of enrichment objects were altered each week over 6 weeks, with a gradual increase in item quantity and placement diversity. The experimental design is illustrated in Fig. 1.

### Euthanasia and removal of the axillary lymph node

At 90 days of age, the animals were euthanized. The procedure was carried out by intraperitoneal injection of a ketamine hydrochloride (0.1 mL/kg) and xylazine (10 mg/kg) mixture. After euthanasia, biological samples were properly collected and stored at − 80 °C until further analyses.

### Bradford assay for protein content

Protein concentration in the axillary lymph node suspension was quantified using the Bradford method^15^. The resulting protein–dye complex exhibits maximum absorbance at 595 nm. Absorbance values are directly proportional to the protein concentration of the analyzed samples, with a 1% bovine serum albumin solution used as the standard.

### Activity of citrate synthase

Citrate synthase is the initial enzyme of the Krebs cycle and is essential for catalyzing the condensation of acetyl-CoA with oxaloacetate to generate citrate. Furthermore, citrate synthase activity is widely used as a biochemical indicator of mitochondrial density and aerobic capacity, as described by Alp et al.^16^. Briefly, the assay was performed in a reaction medium containing Tris–HCl buffer (pH 8.2), magnesium chloride (MgCl_2_), ethylenediaminetetraacetic acid (EDTA), and 5,5′-dithiobis (2-nitrobenzoic acid) (DTNB), which reacts with the free CoA released during the enzymatic reaction, producing a yellow chromophore. The reaction mixture consisted of DTNB (ε = 13.6 μmol/(mL·cm)), 3 μM acetyl-CoA, 5 μM oxaloacetate, and 0.3 mg/mL of tissue homogenate. Enzyme activity was determined by measuring the change in absorbance at 412 nm for 3 min at 25 °C and was expressed as millimoles per minute per milligram of protein (mM/min/mg protein).

## Kinetics of pyridine nucleotide oxidation (NAD^+^/NADH)

The levels of oxidized (NAD +) and reduced (NADH) coenzyme in axillary lymph node were determined by incubating 0.1 mg/ml of protein sample with 50 mM TRIS buffer (pH 7.4) at room temperature for 1 min. Absorbances were measured with a spectrophotometer (IL-592, ELEVE, China) at 260 and 340 nm for NAD + and NADH, respectively. The extent of nicotinamide adenine dinucleotide (NAD^+^/NADH) oxidation was determined from the decay of the fluorescence signal under controlled temperature conditions and stirring. Internal calibration of the system was performed by adding known concentrations of NAD^+^ or NADH to confirm signal accuracy. Results were expressed as μM per mg of protein^1,17^.

### Oxidative balance

#### Thiobarbituric acid reactive substances (TBARS)

Lipid peroxidation was evaluated by quantifying thiobarbituric acid–reactive substances (TBARS) using the colorimetric method described by Buege and Aust^18^. Briefly, aliquots of axillary lymph nodes homogenates were combined with 30% trichloroacetic acid (TCA) and 3 mM Tris–HCl buffer (pH 7.4), followed by centrifugation at 3000 rpm for 10 min. The resulting supernatant was incubated with 0.73% thiobarbituric acid (TBA), which reacts with lipid peroxidation products to form a pink chromogen. The reaction was carried out at 100 °C for 15 min, after which absorbance was measured at 535 nm using a glass cuvette. Results were expressed as millimoles per milligram of protein (mM/mg)^18,19^.

### Protein oxidation assay

Protein carbonyl levels were quantified according to the method described by Reznick and Packer^20^. Briefly, 300 μg of protein were precipitated with 30% (w/v) trichloroacetic acid (TCA) and centrifuged at 1,180 × g for 14 min. The resulting pellet was resuspended in 10 mM 2,4-dinitrophenylhydrazine (DNPH) and incubated at room temperature in the dark for 1 h, with gentle agitation every 15 min. Following incubation, the samples were washed and centrifuged three times using an ethyl acetate/ethanol solution. The final pellet was then dissolved in 6 M guanidine hydrochloride and incubated at 37 °C for 30 min. Absorbance was read at 370 nm, and results were expressed as millimoles per milligram of protein (mM/mg)^20,21^.

### Superoxide dismutase (SOD)

SOD activity was assessed in the axillary lymph nodes using the adrenaline autoxidation assay, which selectively evaluates SOD activity. The reaction was monitored spectrophotometrically at 480 nm. The assay was performed in a 1 mL quartz cuvette containing 0.1 M carbonate buffer (pH 10.2), 0.1 mM EDTA, the sample, and 150 mM adrenaline. Changes in absorbance at 480 nm were recorded for 90 s at 30 °C. Enzyme activity was expressed as millimoles of product formed per minute per milligram of protein (mM/min/mg protein)^11,12,14,22^.

### Catalase

Enzymatic activity was determined in the axillary lymph nodes by measuring the rate of hydrogen peroxide (H₂O₂) breakdown, followed by a reduction in absorbance at 240 nm at 20 °C. The reaction was initiated by adding the biological sample to a solution containing 50 mM phosphate buffer (pH 7.0) and 0.3 mM H₂O₂. Absorbance readings were continuously collected for 3 min, and enzyme activity was calculated and expressed as millimoles of H₂O₂ consumed per minute per milligram of protein (mM/min/mg protein)^23,24^.

### Glutathione S-transferase (GST)

The activity of glutathione S-transferase (GST) was determined in the axillary lymph nodes using the method described initially by Habig^25^. Assays were carried out in a reaction system containing 0.1 M potassium phosphate buffer (pH 6.5), 1 mM EDTA, 1 mM reduced glutathione (GSH), the sample, and 1 mM 1-chloro-2,4-dinitrobenzene (CDNB). GST activity was quantified by measuring the rate of formation of 2,4-dinitrophenyl-S-glutathione (DNP-SG) at 30 °C through spectrophotometric detection at 340 nm. Enzyme activity was reported as millimoles per minute per milligram of protein (mM/min/mg protein)^11,12,14,25^.

### Non-enzymatic antioxidant response

Reduced glutathione (GSH) concentrations were quantified in the axillary lymph nodes following the procedure described by Hissin and Hilf. Samples were initially diluted 1:10 in 0.1 M phosphate buffer supplemented with 5 mM EDTA (pH 8.0) and subsequently incubated with o-phthaldialdehyde (OPT; 1 mg/mL) at room temperature for 15 min. Fluorescence readings were obtained using excitation and emission wavelengths of 350 nm and 420 nm, respectively. Oxidized glutathione (GSSG) levels were measured after treating the samples with 40 mM N-ethylmaleimide for 30 min at room temperature, followed by the addition of 100 mM NaOH. Data were expressed as micromoles per milligram of protein (µM/mg protein), and cellular redox status was evaluated by calculating the GSH/GSSG ratio^26,27^.

### Sulfhydryl’s

Total and protein sulfhydryl group levels were quantified using the method described by Aksenov and Markesbery^28^. In this assay, thiol groups react with 5,5′-dithiobis (2-nitrobenzoic acid) (DTNB), forming a yellow chromophore. The reaction was carried out in homogenates containing 200 µg of protein, and absorbance was measured spectrophotometrically at 412 nm. Results were expressed as millimoles per milligram of protein (mM/mg protein)^13,28^.

### Cytokine levels in serum

Cytokine concentrations were measured using the BD™ Cytometric Bead Array (CBA) system (BD Biosciences, USA) for the detection of interleukin-2 (IL-2), interleukin-4 (IL-4), interleukin-6 (IL-6), interleukin-10 (IL-10), interleukin-17A (IL-17A), interferon-gamma (IFN-γ), and tumor necrosis factor-alpha (TNF-α). Serum samples were thawed and diluted 1:2 (v/v) with assay diluent, and the CBA assay was conducted in accordance with the manufacturer’s instructions (BD Pharmingen™). Data acquisition was carried out using a BD Accuri™ C6 flow cytometer^13^.

### Statistical analysis

Data normality was evaluated using the Shapiro–Wilk test. Results were initially presented as mean ± standard deviation. Comparisons between two experimental groups were conducted using either the unpaired Student’s t test or the Mann–Whitney test, as appropriate. Effect size was calculated using Hedges’ g, based on the group means, standard deviations, and post-intervention sample sizes. Effect size magnitude was interpreted as negligible (0.0 to < 0.2), small (≥ 0.2 to < 0.5), moderate (≥ 0.5 to < 0.8), large (≥ 0.8 to < 1.3), or very large (≥ 1.3), Supplementary Table 1. Statistical significance was defined as p < 0.05. All statistical analyzes were performed using GraphPad Prism version 10 (GraphPad Software Inc., La Jolla, CA, USA).

## Results

### Mitochondrial indicators

**Fig. 2:**
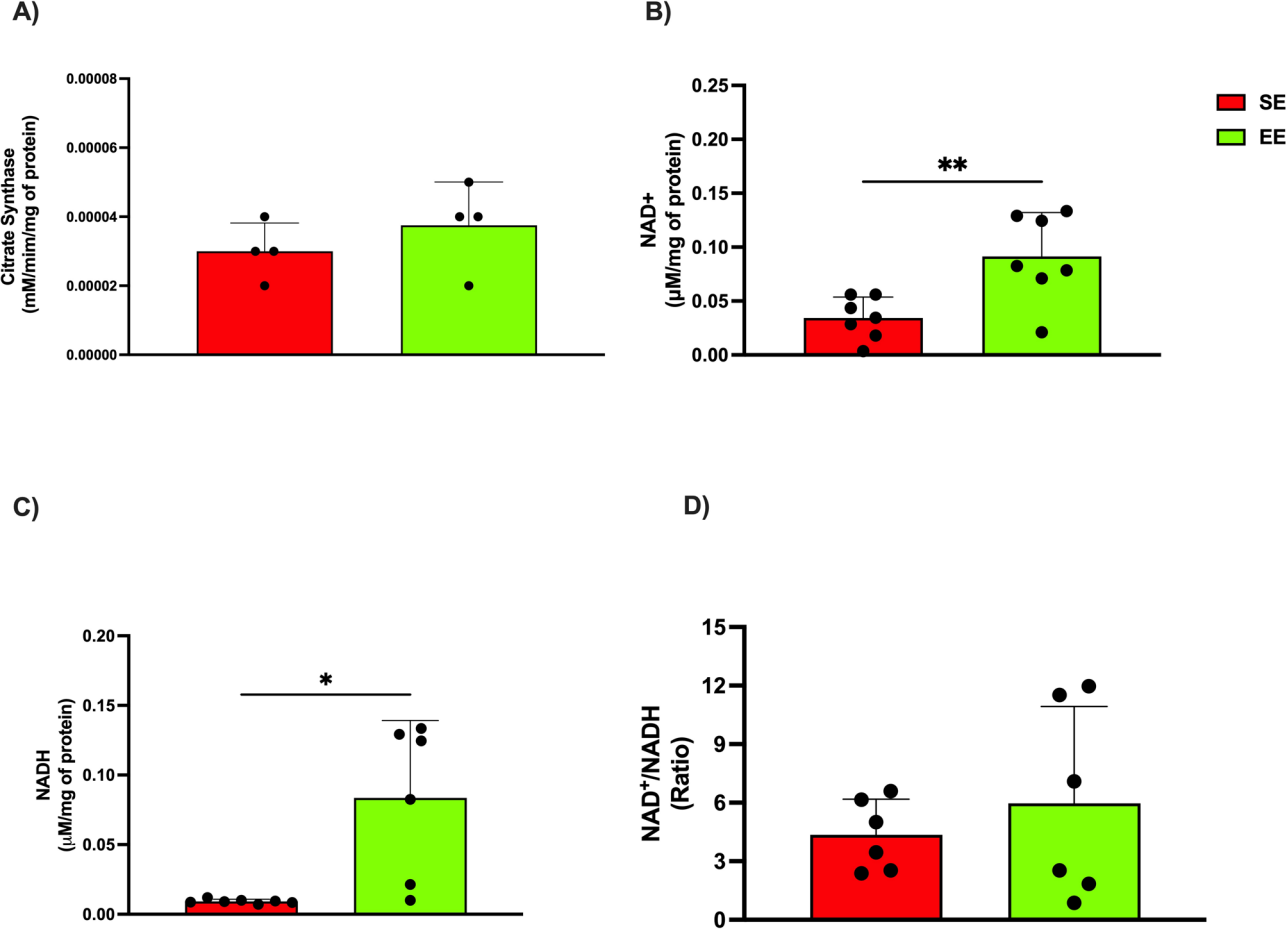
Mitochondrial metabolism markers in the axillary lymph nodes of juvenile male C57BL/6 mice after 6 weeks of Environmental Enrichment. (**A**) Citrate synthase activity; (**B**) NAD^+^ levels; (**C**) NADH levels; and (D) NAD^+^/NADH ratio. Data are expressed as mean ± SD (n = 4–7 per group). *p < 0.05; **p < 0.01; ***p < 0.001. Differences were assessed using the unpaired Student’s *t*-test or the Mann–Whitney test. EE: Environmental Enrichment; SE: Standard Environment.

Initially, citrate synthase activity was evaluated; this enzyme catalyzes the condensation of acetyl-CoA with oxaloacetate and is a key component of the Krebs cycle. After the EE, no significant differences were observed between the groups (p = 0.36), Fig. 2A. We assessed the levels of NAD + and NADH, which act as coenzymes involved in energy metabolism as electron acceptors. These indicators relate to mitochondrial function efficiency. After 6 weeks, we observed significant increases in NAD^+^ (p = 0.0009) and NADH (p = 0.01) levels in the animals that underwent EE, when compared to the SE group, Fig. 2B,C. Finally, when analyzing the NAD + /NADH ratio, we observed no significant differences between the groups (p = 0.46; Fig. 2D).

### Oxidative stress markers and antioxidant enzymatic defenses

**Fig. 3:**
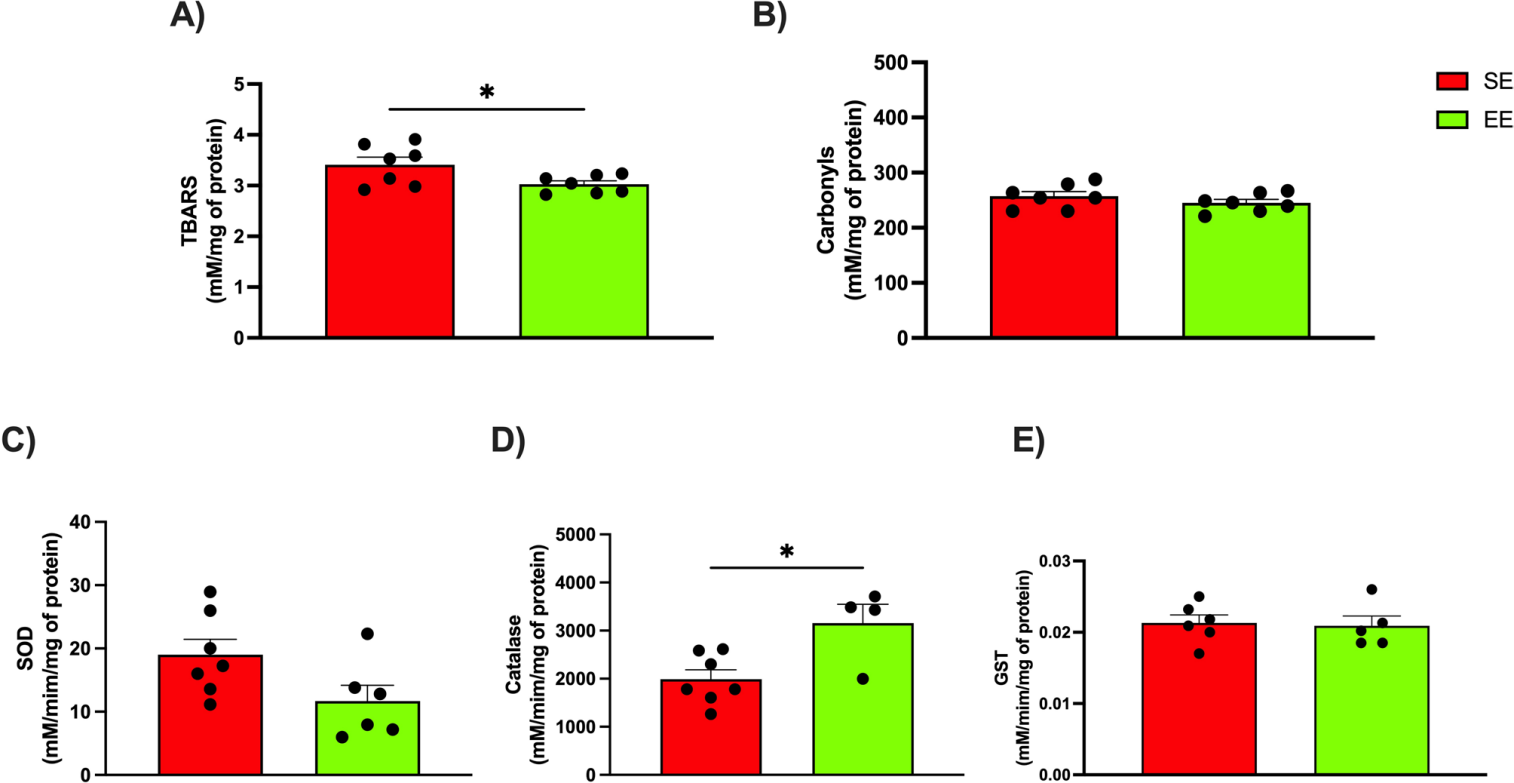
Redox balance parameters in the axillary lymph nodes of juvenile male C57BL/6 mice after 6 weeks of Environmental Enrichment. (A) Thiobarbituric Acid Reactive Substances (TBARS); (**B**) Carbonyls; (**C**) Superoxide Dismutase (SOD); (**D**) Catalase; and (**E**) Glutathione S-Transferase (GST). Data are expressed as mean ± SD (n = 4–7 per group). *p < 0.05. Differences were assessed using the unpaired Student’s *t*-test or the Mann–Whitney test. EE: Environmental Enrichment; SE: Standard Environment.

We evaluated the levels of thiobarbituric acid-associated reactive substances (TBARS) and carbonyls, which are classic markers linked to oxidative stress. First, we assessed TBARS levels, which reflect lipid oxidation induced by oxygen or nitrogen free radicals; this phenomenon is called lipid peroxidation. After 6 weeks, we observed a significant decrease in TBARS in animals exposed to EE compared with the SE group (p = 0.04; Fig. 3A). Next, we assessed levels of carbonyls linked to protein oxidation, resulting from free-radical damage. Our data did not identify significant differences between the groups (p = 0.26; Fig. 3B).

On the other hand, we evaluated three components of the enzymatic antioxidant defense system, namely SOD, catalase, and GST. Biologically, SOD is responsible for transforming the superoxide radical into hydrogen peroxide (H_2_O_2_). In line with this process, catalase converts hydrogen peroxide into oxygen and water, preventing cellular damage. Finally, GST participates in cellular detoxification by catalyzing the conjugation of GSH to toxic compounds, thereby reducing their toxicity and facilitating their elimination.

The data did not show significant differences between the groups in SOD (p = 0.06) and GST (p = 0.82) activity (Fig. 3C,D). However, when analyzing catalase, we found that animals exposed to EE for 6 weeks showed a significant increase in this marker compared with the SE group.

### Non-enzymatic antioxidant compounds

Next, to comprehensively understand the effects of EE on oxidative balance, we analyzed levels of GSSG, GSH, the redox state, and sulfhydryl groups. GSSG is formed when two molecules of GSH donate electrons to neutralize free radicals, resulting in the formation of a disulfide bond. GSH, in turn, acts as a primary detoxifying agent within the cellular environment and plays a crucial role in maintaining redox homeostasis. The balance between these two forms, expressed as the GSH/GSSG ratio, is widely recognized as a reliable indicator of the cellular redox state. Closely associated with this system are sulfhydryl, or thiol, groups (–SH), which contribute to the structural stability of macromolecules such as proteins and to the regulation of cellular oxidation–reduction reactions. Through their interaction with GSH, thiol groups are maintained in their reduced state. Additionally, this system is essential for neutralizing peroxides via glutathione peroxidase (GPx).

**Fig. 4:**
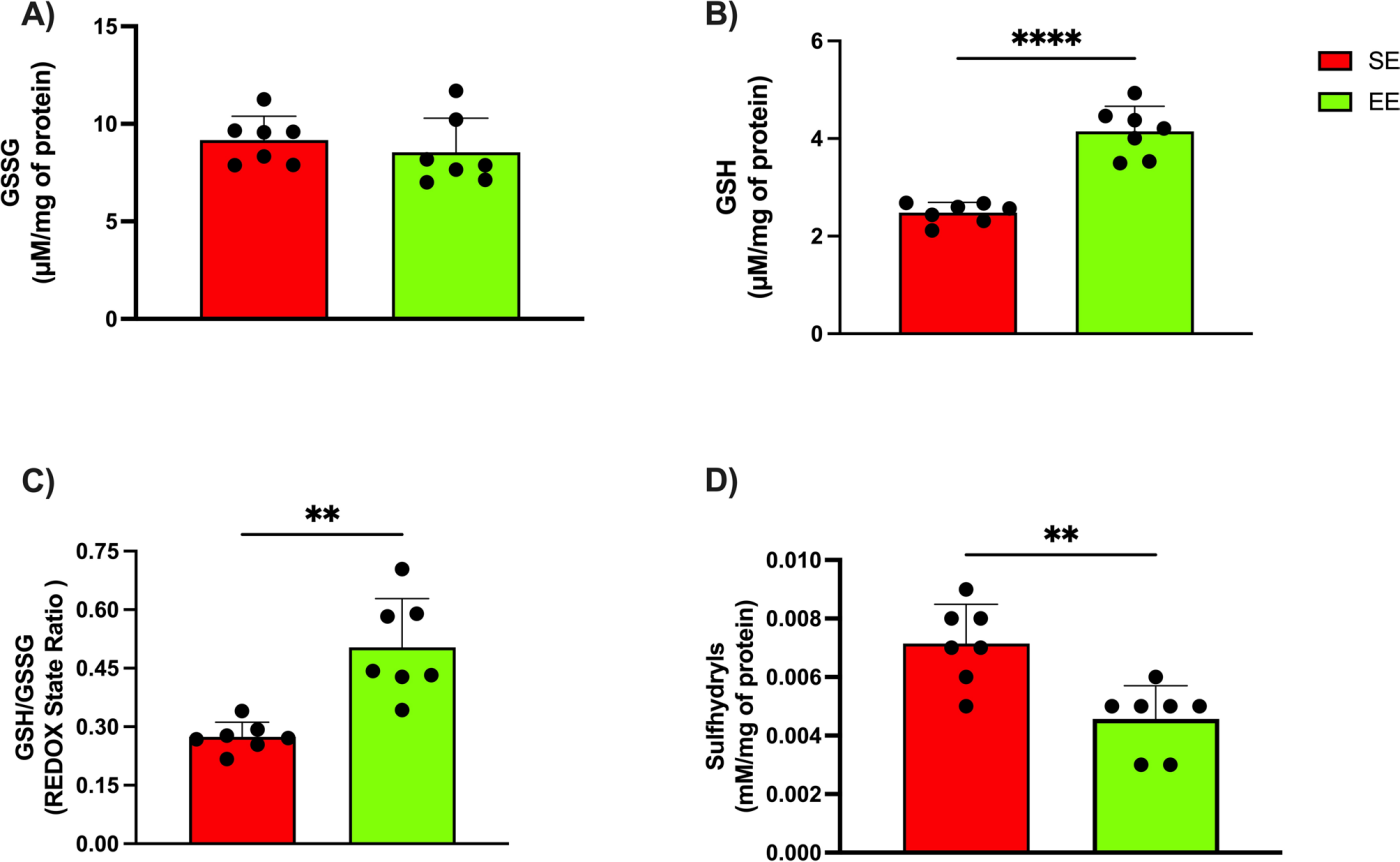
Assessment of non-enzymatic antioxidant compounds; (**A**) Oxidized glutathione (GSSG); (**B**) Reduced glutathione (GSH); (**C**) Redox state (GSH/GSSG ratio); and (**D**) Sulfhydryl groups in the axillary lymph nodes of juvenile male C57BL/6 mice after 6 weeks of Environmental Enrichment. Data are expressed as mean ± SD (n = 7 per group). **p < 0.01; ****p < 0.0001. Differences were assessed using the unpaired Student’s *t*-test or the Mann–Whitney test. EE: Environmental Enrichment; SE: Standard Environment.

After 6 weeks, We did not observe significant differences between the groups, p = 0.45, (Fig. 4A). Additionally, we observed a significant increase in GSH levels (p < 0.0001) and in the GSH/GSSG ratio (p = 0.002) in the EE group compared to the SE animals (Fig. 4B,C). On the other hand, sulfhydryl levels in animals exposed to EE decreased significantly (p = 0.006) compared to the SE group (Fig. 4D).

### Cytokines linked to the Th1/Th2 profile

**Fig. 5:**
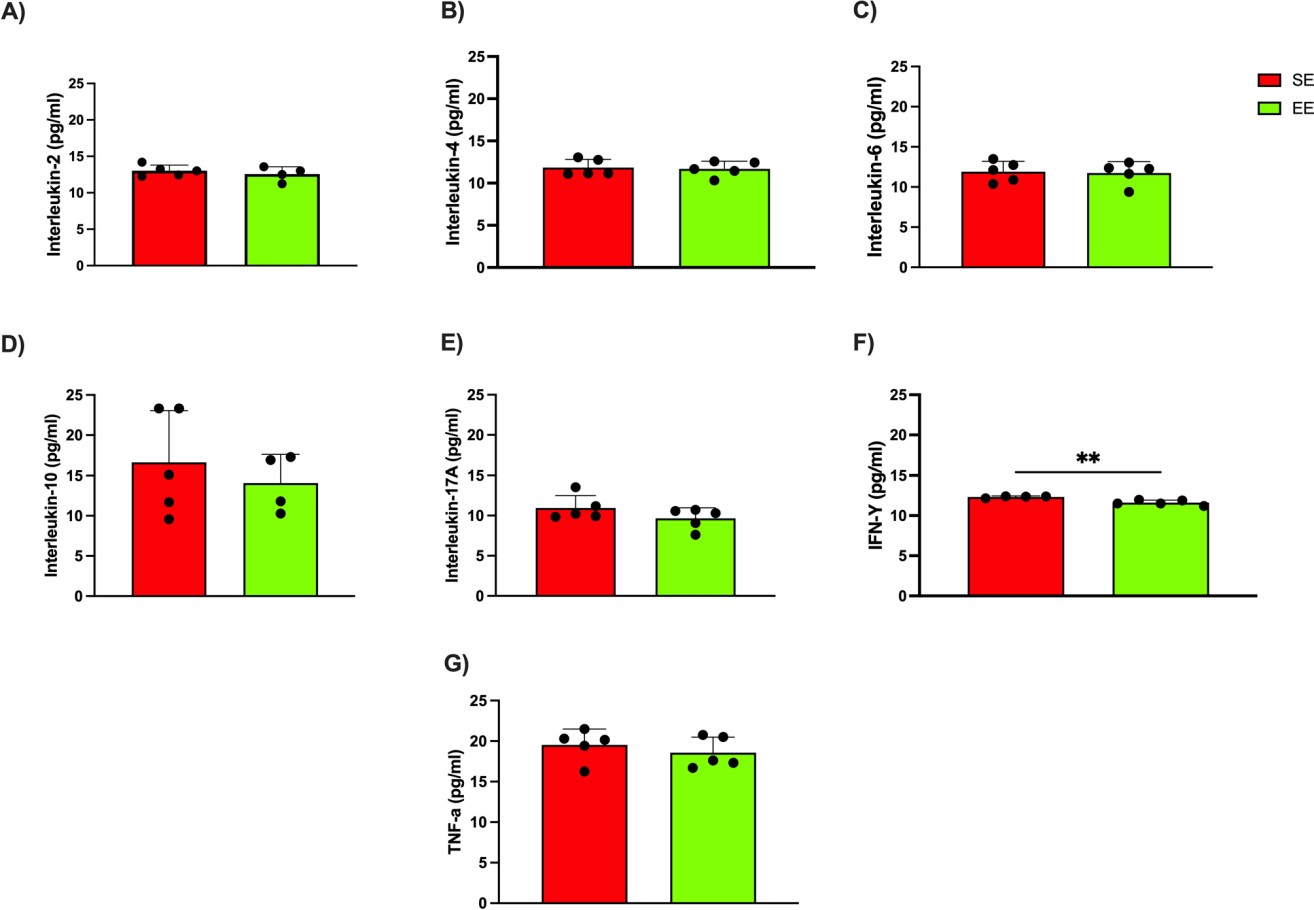
Effect of Environmental Enrichment on serum cytokine levels linked to the Th1/Th2 profile in adult male C57BL/6 mice; (**A**) Interleukin-2 (IL-2); (**B**) Interleukin-4 (IL-4); (**C**) Interleukin-10 (IL-10); (**D**) Interleukin-6 (IL-6); (**E**) Interleukin-17A (IL-17A); (**F**) Interferon gamma (IFN-γ); and (**G**) Tumor Necrosis Factor Alpha (TNF-α). Data are expressed as mean ± SD (n = 4–5 per group). *p < 0.05. Differences were assessed using the unpaired Student’s *t*-test or the Mann–Whitney test. EE: Environmental Enrichment; SE: Standard Environment.

At the serum level, aiming to observe a systemic impact of EE, we analyzed the levels of cytokines related to the Th1/Th2 lymphocyte profile, namely IL-2, IL-4, IL-6, IL-10, IL-17A, IFN-γ, and TNF-α. The Th1/Th2 profile reflects a fundamental pattern of the adaptive immune response, characterized by the type of activated CD4^+^ helper T lymphocyte and the cytokines it produces. Our data revealed no significant differences between the groups in the levels of IL-2 (p = 0.48), IL-4 (p = 0.95), IL-6 (p = 0.83), IL-10 (p = 0.47), IL-17A (p = 0.19), or TNF-α (p = 0.46) after 6 weeks of EE, Fig. 5A–E and G, respectively. However, when we evaluated IFN-γ levels, we identified a significant decrease (p = 0.003) in animals exposed to EE for 6 weeks compared to the SE group (Fig. 5F).

## Discussion

Several studies have demonstrated the beneficial effects of EE in modulating behavior, including depression-, fear-, and anxiety-like phenotypes, as well as improving neurodegenerative and neuropathic conditions^2,3,29^. Herein, the present study demonstrates that EE induces a coordinated redox and metabolic adaptation in lymph nodes, characterized by improved mitochondrial redox efficiency, reduced oxidative damage, selective reinforcement of antioxidant defenses, and subtle but functionally relevant immunomodulatory effects. Importantly, these physiological adaptations occur in the absence of evident changes in mitochondrial mass or broad alterations in systemic cytokine profiles, indicating that EE primarily promotes qualitative immunometabolic optimization rather than generalized immune activation or suppression.

Citrate synthase activity, a surrogate marker of mitochondrial content, remained unchanged following EE exposure. This finding suggests that mitochondrial content was not substantially modified; however, given the non-specific nature of this marker, no definitive conclusions can be drawn regarding mitochondrial biogenesis^30^. Instead, the significant increases in NAD^+^ and NADH levels, in the absence of changes in the NAD^+^/NADH ratio, suggest an expansion of the total pyridine nucleotide pool rather than a shift in redox balance^31^. Moreover, NAD^+^ serves as a critical substrate for NAD^+^-dependent enzymes such as sirtuins, which integrate mitochondrial metabolism, antioxidant defenses, and immune regulation^32,33^. Thus, EE appears to enhance mitochondrial metabolic capacity in lymph nodes by increasing the availability of redox cofactors, supporting a more metabolically flexible and functionally efficient state rather than structural remodeling.

Consistent with this interpretation, EE markedly reduced lipid peroxidation, as indicated by lower TBARS levels, reflecting diminished oxidative damage to membrane lipids. Lipid peroxidation is particularly detrimental in immune tissues, where membrane integrity is essential for receptor signaling, antigen presentation, and intercellular communication^34,35^. In contrast, protein carbonyl levels remained unchanged, suggesting that EE does not induce widespread or irreversible oxidative protein damage. This selective protection of membrane lipids indicates a controlled redox adaptation rather than a generalized suppression of oxidative processes.

At the level of enzymatic antioxidant defenses, EE induced a selective increase in catalase activity without altering superoxide dismutase (SOD) or glutathione S-transferase (GST) activities. This pattern suggests that EE does not elicit a broad antioxidant stress response but instead fine-tunes specific components of the antioxidant network. Catalase plays a central role in detoxifying hydrogen peroxide generated during mitochondrial respiration and immune cell activation, thereby preventing hydroxyl radical formation^1,11,23^. Its selective upregulation, in the absence of increased SOD activity, implies reduced superoxide generation upstream and enhanced hydrogen peroxide clearance downstream. This targeted adjustment is consistent with improved mitochondrial efficiency and controlled ROS signaling, a hallmark of physiological redox optimization rather than oxidative challenge.

Complementing these enzymatic changes, EE remodeled the non-enzymatic antioxidant system. Lymph nodes from EE-exposed animals exhibited increased levels of reduced glutathione (GSH), and a significantly elevated GSH/GSSG ratio, collectively indicating a shift toward a more reduced and protective intracellular environment. This glutathione-dependent redox buffering enhances peroxide detoxification, preserves protein thiol homeostasis, and supports immune cell metabolic fitness^36,37^. Interestingly, total free sulfhydryl levels were reduced despite the improved glutathione redox state. This apparent paradox likely reflects increased utilization of thiol groups in redox-sensitive signaling pathways, antioxidant reactions, and glutathione-dependent processes rather than increased oxidative damage. Such dynamic engagement of thiols is increasingly recognized as a feature of healthy redox signaling and cellular adaptability^38^.

Mechanistically, these redox adaptations are consistent with the activation of conserved antioxidant pathways, including the Nuclear factor erythroid 2–related factor 2 (NRF2) signaling pathway. EE and increased locomotor activity have been shown to activate NRF2 in multiple tissues, leading to transcriptional upregulation of antioxidant enzymes and improved mitochondrial homeostasis^39,40^. In lymph nodes, NRF2-mediated regulation is particularly relevant, as immune cells require tightly controlled redox signaling to balance proliferation, activation, and cytokine production^41^. The combination of reduced lipid peroxidation, selective catalase induction, and enhanced glutathione redox balance observed here aligns with an NRF2-driven response designed to limit hydrogen peroxide accumulation while preserving physiological ROS-dependent signaling.

Despite these pronounced redox and metabolic adaptations within lymph nodes, EE did not alter most circulating cytokines associated with Th1, Th2, Th17, or regulatory responses, indicating that EE does not induce systemic immune activation or suppression under basal conditions. Notably, the levels of IFN-γ, a key Th1 cytokine involved in macrophage/microglia activation and pro-inflammatory signaling, were selectively reduced, suggesting a more restrained immune tone^42^. This finding closely mirrors previous observations showing that T cells derived from EE-exposed animals produce less IFN-γ upon stimulation while maintaining normal production of other cytokines^6^. Given the strong coupling between redox state, NAD^+^-dependent signaling, and T cell effector programming, the redox-optimized lymph node milieu induced by EE likely contributes to restrained IFN-γ production by modulating activation thresholds rather than lineage commitment^36,43,44^.

Collectively, these findings support a model in which EE promotes immunometabolic homeostasis within lymph nodes through coordinated modulation of mitochondrial redox status, NAD^+^ availability, NRF2-dependent antioxidant defenses, and glutathione redox buffering. These adaptations establish a redox-optimized microenvironment that converges on T cells, where improved redox control and NAD^+^-dependent signaling restrain excessive Th1-type inflammatory output while preserving immune competence. From a broader perspective, this mechanism is particularly relevant to aging and inflammatory diseases, conditions characterized by declining NAD^+^ levels, impaired antioxidant capacity, chronic oxidative stress, and dysregulated immune activation^9,45,46^. By restoring redox balance and enhancing metabolic resilience, EE emerges as a powerful non-pharmacological strategy to fine-tune immune function through metabolic and redox pathways.

## Limitations and strengths

Besides, this study, for the first time, presents analyses of EE and basal redox balance in lymph nodes under non-pathological conditions. First, we use only male C57BL/6 mice, which restricts the extrapolation of the findings to females, given known sex differences in immune regulation, mitochondrial metabolism, and redox biology. Given this limitation, other studies are being conducted with female mice, and we hope to have more data soon on the effects of EE in females. Second, analyses were limited to axillary lymph nodes, and other secondary lymphoid organs were not evaluated, preventing conclusions about whether the observed redox adaptations represent a systemic immunological phenomenon. Also, keep this limitation in mind: we are conducting parallel additional studies using additional immune tissues. Third, cytokine measurements were restricted to basal serum levels, and no immune challenge or ex vivo stimulation assays were conducted, limiting the ability to draw more robust conclusions about functional immune responsiveness. Fourth, EE exposure was restricted to 6 weeks, which represents an important limitation of the present study. Previous studies have demonstrated that the duration of EE critically influences biological outcomes, including behavioral and neuroplastic adaptations^47,48^. Future studies incorporating multiple time points will be necessary to define the temporal dynamics of EE-induced immunometabolic adaptations.

Although the present study has important strengths, its primary strength is the comprehensive evaluation of redox homeostasis in lymph nodes, integrating markers of oxidative damage, enzymatic and non-enzymatic antioxidant systems, and pyridine nucleotide status. This integrated approach allows a robust assessment of redox homeostasis rather than reliance on isolated biomarkers. Additionally, the focus on lymph nodes, which are central to adaptive immune activation, addresses a significant gap in EE research, which has largely emphasized neural or peripheral metabolic tissues. The use of standardized EE protocols, rigorous biochemical methodologies, and effect size analyses enhances the reliability and interpretability of the findings. Importantly, the selective nature of the adaptations—improved antioxidant buffering, reduced lipid peroxidation, and expansion of the pyridine nucleotide pool without changes in the NAD^+^/NADH ratio—supports the conclusion that EE promotes physiological immunometabolic optimization. This specificity increases the translational relevance of EE as a non-pharmacological strategy promoting immune tissue resilience.

## Conclusion

Taken together, these results indicate that EE fosters a redox-optimized and metabolically robust lymph node microenvironment, characterized by increased antioxidant buffering capacity and expansion of the pyridine nucleotide pool without alterations in the NAD^+^/NADH ratio, in the absence of extensive immune suppression or activation. These adaptive responses underscore EE as a powerful non-pharmacological approach for modulating immunometabolic homeostasis, with potential implications for immune conditions associated with oxidative stress, including aging, cancer, and neurodegenerative disorders.

## Supplementary Information

Below is the link to the electronic supplementary material.

Supplementary Material 1

## Data Availability

The data presented in this study are available upon request from the corresponding author of this paper. The data are not publicly available for further publication because of restrictions related to the ongoing work.
