# Supplementary material for: Effects of environmental enrichment on lymph node mitochondrial-redox status and immune response in adult male C57BL/6 mice
Source: Sci Rep. 2026 Jun 3;16:25235. doi: 10.1038/s41598-026-55690-8 (PMC13470239; doi:10.1038/s41598-026-55690-8)
Supplement: Supplementary file 1 [file 41598_2026_55690_MOESM1_ESM.docx]

| ***Supplementary Table 1.*** Effect of environmental enrichment on axillary lymph node mitochondrial-REDOX status and serum immune response in adult male C57BL/6 mice | | | |
| --- | --- | --- | --- |
| **Variable** | **SE (Mean with SD)** | **EE (Mean with SD)** | ***g*** |
| TBARS | 3.411 ± 0.39 | 3.027 ± 0.17 | **-1.27** |
| Carbonlys | 257.1 ± 22.0 | 245.0 ± 16.5 | -0.62 |
| Superoxide Dismutase | 19.00 ± 6.47 | 11.68 ± 6.09 | **-1.16** |
| Catalase | 1991 ± 515.1 | 3157 ± 780 | **1.89** |
| Gluthatione S Transferase | 0.0027 ± 0.002 | 0.020 ± 0.003 | -0.28 |
| Oxidized Gluthatione | 9.171 ± 1.220 | 8.540 ± 1.775 | -0.40 |
| Reduced Gluthatione | 2.484 ± 0.207 | 4.146 ± 0.516 | **4.22** |
| REDOX State | 0.274 ± 0.03 | 0.503 ± 0.12 | **2.61** |
| Sulfhydryls | 0.007 ± 0.001 | 0.004 ± 0.001 | **-3.00** |
| Citrate Synthase | 0.00003 ± 0.000008 | 0.000037 ± 0.000012 | 0.68 |
| NAD^+^ | 0.034 ± 0.01 | 0.103 ± 0.02 | **4.36** |
| NADH | 0.009 ± 0.001 | 0.031 ± 0.035 | **0.93** |
| NAD^+^/NADH | 4.350 ± 1.82 | 5.973 ± 4.95 | 0.43 |
| Interleukin-2 | 13.03 ± 0.76 | 12.58 ± 1.00 | 0.51 |
| Interleukin-4 | 11.84 ± 0.97 | 11.68 ± 0.90 | -0.17 |
| Interleukin-6 | 11.92 ±1.28 | 11.74 ± 1.41 | -0.13 |
| Interleukin-10 | 16.61 ± 6.44 | 14.07 ± 3.54 | -0.47 |
| Interleukin-17A | 10.94 ± 1.54 | 9.65 ± 1.30 | **-0.90** |
| Interferon Gamma | 12.33 ± 0.11 | 11.61 ± 0.31 | **-2.93** |
| Tumor Necrosis Factor Alpha | 19.53 ± 1.96 | 18.58 ± 1.90 | -0.49 |

**Legend:** NAD+: Nicotinamide Adenine Dinucleotide; NADH: Reduced Nicotinamide Adenine Dinucleotide, TBARS: Thiobarbituric Acid Reactive Substances.
